# Supplementary material for: Using geographically weighted regression analysis to assess predictors of home birth hot spots in Ethiopia
Source: PLoS One. 2023 Jun 6;18(6):e0286704. doi: 10.1371/journal.pone.0286704 (PMC10243638; doi:10.1371/journal.pone.0286704)
Supplement: Attachment — Submitted filename: Reviewers comment.docx [file pone.0286704.s001.docx]

**Major general concern**

From the spatial analysis, it is obvious the analysis was done at the regional level; however, the authors did not include any regional level variables in the study. But instead, an aggregation of the individual-level variables were used. How did the authors control the heterogeneity of the various enumeration areas making up a region? Also, the authors must read **the concept of ecological fallacy** and indicate how they corrected that in their study? Because they used individual-level aggregated data to represent the regions.

**Background**

The background of the manuscript is well written and clearly articulates the burden of home birth. However, there is not enough scientific justification besides the authors indicating that spatial regression considers non-stationary variables. Furthermore, despite indicating that the manuscript accounts for non-stationary variables, the authors failed to state what non-stationary variables were included in their study and how different they are from previous studies.

**Methods**

the outcome variable considered in the study is a dichotomised variable based on its description; the authors failed to indicate how they performed a GWR, which takes a continuous dependent variable and continuous independent variables.

The list of explanatory variables appears limited since the variables influencing home birth go beyond the individual and household variables. In addition, the authors failed to account for variables such as distance to facility, health insurance and cost related to health care utilisation.

The authors didn’t describe how the weighted proportion was estimated and at what level they measured the weighted proportion of home birth.

**Results**

The results presented in Table 2: Summary of ordinary least squares result does not account for all the explanatory variables stated in the methods section. The authors did not provide any justification after stating that all variables were considered.
