# Supplementary material for: Using geographically weighted regression analysis to assess predictors of home birth hot spots in Ethiopia
Source: PLoS One. 2023 Jun 6;18(6):e0286704. doi: 10.1371/journal.pone.0286704 (PMC10243638; doi:10.1371/journal.pone.0286704)
Supplement: Attachment — Submitted filename: PLoSOnereview.docx [file pone.0286704.s002.docx]

Best of luck with this important work. consider working with this free manuscript support service to get it out into the world:

https://sites.google.com/umich.edu/prepss

Intro:

Consider keeping information focused to African countries for this section, unless there is no information about the topic in that region (e.g., is line 98 needed about USA?)

Line 106: there is a wealth of literature on socio-cultural factors that influence home birth in sub-Saharan Africa. A few should be included here.

Lines 111-116: consider stating the primary and secondary aims of your study here.

Methods:

Line 131=132: this sentence is confusing, I’m not sure what it means- consider omitting it

Spatial regression section: Much of this is not necessary and could be included in an appendix. Rather than explain the method itself, only keep in the text what you did in your analysis ( do not need to explain the method itself).

Table 1.

I assume this is the frequency and percent? So Add N(%) to the column headings

Might want to consider doing regression here with urban/rural as the predictor and homebirth as the outcome, rather than just listing frequencies and percentages

Lines 202-210: If this section begins your results, then have a heading that says Results above it to orient the reader

Please omit explanatory sentences about the methods use and stick to just reporting the results here. Further for lines 211-214, need to include the results in the text to match Table 2 for each sociodemographic that is listed.

For line 215: need to put results right next to each variable you are reporting on (e.g., rural resident (result here) )

Table 2: do not need to include OLS diagnosis- this could be included in appendices but not necessary

Line 231: Please include the results in text adjacent to each variable.
