# Supplementary material for: Using geographically weighted regression analysis to assess predictors of home birth hot spots in Ethiopia
Source: PLoS One. 2023 Jun 6;18(6):e0286704. doi: 10.1371/journal.pone.0286704 (PMC10243638; doi:10.1371/journal.pone.0286704)
Supplement: Attachment — Submitted filename: PLoSOnereview.docx [file pone.0286704.s004.docx]

Using a geographic approach to identify ‘hotspots’ of homebirth is a novel approach, and addresses a major problem in maternal health.

However, this manuscript requires further edits with attention to grammar and clarity.

Of greater importance, although using a novel structural lens to view homebirth in Ethiopia, the focus remains on individual characteristics of women. Might there be data that could be merged with the publicly available data that was used that could identify distance to facilities, concentration of schools, funding for roads, etc.?

It is problematic to frame this as a problem based on individual women’s characteristics, but say the target audience is policy makers etc. What are the structural factors that could be addressed, and how can the authors include those in their analysis/results/discussion?

Please consider working with the University of Michigan’s free of charge publication support team:

Pre-publication Support Service

<https://sites.google.com/umich.edu/prepss>

To get this manuscript ready for submission.
