# Supplementary material for: Using geographically weighted regression analysis to assess predictors of home birth hot spots in Ethiopia
Source: PLoS One. 2023 Jun 6;18(6):e0286704. doi: 10.1371/journal.pone.0286704 (PMC10243638; doi:10.1371/journal.pone.0286704)
Supplement: Attachment — Submitted filename: Point by point response.docx [file pone.0286704.s005.docx]

**To: PLOS ONE**

From: Samuel Hailegebreal

Subject: **A letter Accompanying Revision in Response to Editors and Reviewer Comments**

**Dear Editors**

The authors would like to thank the editorial team and team of reviewers for constructive and valuable comments. The authors are very happy to submit the revised version of the manuscript entitled **“Using geographically weighted regression analysis to assess predictors of home birth hot spots in Ethiopia”** for its publication in your Journal. The comments of the editors and the reviewers were highly insightful and enabled us to greatly improve the quality of our manuscript. In this revised manuscript we made substantial changes to address your concerns in a point-by-point response. We are very keen to incorporate further comments, if any, for the betterment of the final manuscript.

**Point by Point Response to - Editor Comments**

1) Please review your reference list to ensure that it is complete and correct. If you have cited papers that have been retracted, please include the rationale for doing so in the manuscript text, or remove these references and replace them with relevant current references. Any changes to the reference list should be mentioned in the rebuttal letter that accompanies your revised manuscript. If you need to cite a retracted article, indicate the article’s retracted status in the References list and also include a citation and full reference for the retraction notice.

Response: we change reference 6,7,10 and also we added 29 and 30 in revised manuscript

**Point by Point Response to Reviewers**

**Reviewer #1**

1. Line 102 -108 have different font types

Response: we corrected in the revised version

2. There are inherent grammatical errors

Response: we edited carefully spelling and grammatical error and also we used the link provided by reviewer Pre-publication Support Service

3. The authors stated “a lack of modeling of the spatial relationships" this statement indicated that they didn't do an extensive literature search.

Response: I appreciate your comment. There are few studies on spatial distribution home delivery in Ethiopia. Yet, according to our search, no study on the place of delivery in Ethiopia has ever used geographically weighted regression.

4. The authors failed to mention the non-stationary variables which are being included in the study

Response: I appreciate your input. Since the EDHS 2019 is interim significant characteristics were not included, this was highlighted in the limitation section.
